# Mfsd2a Reverses Spatial Learning and Memory Impairment Caused by Chronic Cerebral Hypoperfusion via Protection of the Blood–Brain Barrier

**DOI:** 10.3389/fnins.2020.00461

**Published:** 2020-06-16

**Authors:** Changhua Qu, Hao Song, Jun Shen, Linling Xu, Yaqing Li, Chujie Qu, Tian Li, Junjian Zhang

**Affiliations:** Department of Neurology, Zhongnan Hospital, Wuhan University, Wuhan, China

**Keywords:** vascular cognitive impairment, chronic cerebral hypoperfusion, blood–brain barrier, major facilitator superfamily domain-containing protein 2a, vesicular transcytosis

## Abstract

Disruption of the blood–brain barrier (BBB) can lead to cognitive impairment. Major facilitator superfamily domain-containing protein 2a (Mfsd2a) is a newly discovered protein that is essential for maintaining BBB integrity. However, the role of Mfsd2a in vascular cognitive impairment has not been explored yet. In this study, a rat model of chronic cerebral hypoperfusion (CCH) was established by producing permanent bilateral common carotid artery occlusion (2VO) in rats. We found that after the 2VO procedure, the rats exhibited cognitive impairment, showed increased BBB leakage within the hippocampus, and had reduced expression of the Mfsd2a protein. The overexpression of Mfsd2a in the rat hippocampus reversed these changes. Further investigations using transmission electron microscopy revealed a significantly increased rate of vesicular transcytosis in the BBB of the hippocampus of the CCH rats; the rate reduced after overexpression of Mfsd2a. Moreover, Mfsd2a overexpression did not cause changes in the expression of tight junction-associated proteins and in the ultrastructures of the tight junctions. In conclusion, Mfsd2a attenuated BBB damage and ameliorated cognitive impairment in CCH rats, and its protective effect on the BBB was achieved via inhibition of vesicular transcytosis.

## Introduction

Vascular cognitive impairment (VCI) and Alzheimer’s disease are major medical issues that affect the health of the elderly population (Yang Y. et al., 2017). Chronic cerebral hypoperfusion (CCH) is the common pathophysiological state underlying both conditions (Zhao and Gong, 2015; Shen et al., 2016).

The blood–brain barrier (BBB) is essential for maintaining the stability of the brain microenvironment. Damage to the BBB is an early pathophysiological factor in many diseases involving brain injury (Daneman and Prat, 2015; Liebner et al., 2018). Previous studies found that BBB damage occurred in the early stage of CCH in rat models (Chen et al., 2015; Yin et al., 2015). In addition, disruption of the BBB caused further structural and functional damage to the brain (Chen et al., 2015; Yin et al., 2015), whereas protective measures targeting the BBB alleviated cognitive impairment in CCH rats (Edrissi et al., 2016; Lee et al., 2017). Therefore, BBB damage is considered a key factor in CCH-induced cognitive impairment (Ueno et al., 2016).

The BBB is maintained due to two properties of the brain microvascular endothelium: the continuous tight junctions and the extremely low rate of vesicular transcytosis (Haseloff et al., 2015). It was previously believed that BBB damage was primarily due to the destruction of tight junctions (Siegenthaler et al., 2013). The role that vesicular transcytosis played has been overlooked, and therefore, there is a lack of research on its influence on BBB damage.

Major facilitator superfamily domain-containing protein 2a (Mfsd2a) is a member of the major facilitator superfamily, and it plays a vital role in post-starvation liver metabolism and development of placental syncytiotrophoblast cells (Angers et al., 2008; Toufaily et al., 2013). Mfsd2a is critical for proper barrier function of the BBB, as suggested by recent studies (Ben-Zvi et al., 2014; O’Brown et al., 2019). Mfsd2a suppresses the formation of caveolae vesicles in the brain microvascular endothelium, thereby maintaining an extremely low rate of vesicular transcytosis in the BBB. It has been demonstrated that BBB permeability is positively correlated with the number of vesicles (Wang et al., 2016; Andreone et al., 2017). Thus, disruption of Mfsd2a expression leads to significantly increased vesicular transcytosis and consequently severe BBB leakage (Ben-Zvi et al., 2014). At present, there is a lack of research on the effects of Mfsd2a in BBB damage and cognitive impairment after CCH.

In this study, we constructed rat models of CCH by performing permanent bilateral common carotid artery occlusion (2VO) surgery and evaluated the changes in Mfsd2a expression and vesicular transcytosis in the BBB. We also investigated the effects and mechanisms of Mfsd2a modulation on BBB damage and cognitive impairment in the CCH rats.

## Materials and Methods

### Animals

Adult male Sprague-Dawley rats (180–200 g) were housed in a climate-controlled room (22 ± 2°C with a 12-h light/dark cycle and a relative humidity of 55 ± 5%) and had access to food and water *ad libitum*. The experimental protocols were approved by the Animal Ethics Committee of the Medical School of Wuhan University.

### Experimental Design

Rats were randomly divided into four groups: the sham group (*n* = 68), 2VO group (*n* = 68), 2VO + control adeno-associated virus (AAV) group (*n* = 44), and 2VO + Mfsd2a AAV group (*n* = 44).

The recombinant AAV (AAV2/9-CMV-r-Mfsd2a-3xflag-GFP virus) overexpressing Mfsd2a was delivered via stereotaxic injection to the 2VO + Mfsd2a AAV group and an empty vector (AAV2/9-CMV-GFP control virus, Hanbio Biotechnology Co., Ltd., Shanghai, China) to the 2VO + control AAV group. After 14 days, the rats in the respective groups received either 2VO surgery or sham surgery. The hippocampal blood flow of rats in the 2VO and sham groups (*n* = 6 per group) was measured preoperatively and immediately after surgery by using a laser Doppler flowmeter. On postoperative days 3, 7, 14, and 28, six rats were sacrificed in the sham and 2VO groups to evaluate the changes in Mfsd2a expression in the hippocampus after CCH using western blot. On days 1, 3, 7, 14, and 28 after surgery, the amount of Evans blue (EB) in the hippocampus of rats from the four groups (*n* = 4 per group) was measured using colorimetric analysis. On day 7, western blot was performed to measure the expression of BBB-related proteins, including Mfsd2a, zonula occludens-1 (ZO-1), occludin, and claudin-5 (*n* = 6 per group). Moreover, transmission electron microscopy (TEM) was used to observe the ultrastructures of the hippocampal BBB (*n* = 3 per group). From the 29th day, the spatial learning and memory abilities of rats (*n* = 9 per group) were assessed using the Morris water maze (MWM) test for six consecutive days. Then a novel object recognition (NOR) test was performed to assess the recognition memory abilities of rats (*n* = 9 per group).

### CCH Model

Chronic cerebral hypoperfusion was induced via 2VO surgery as described previously (Xu et al., 2010). Food and water were withheld for 1 day prior to surgery. Rats were anesthetized with 1% Pelltobarbitalum Natricum (40 mg/kg i.p.). The bilateral common carotid arteries were exposed via a midline ventral incision and permanently ligated with a silk suture. Rats receiving the sham operation were treated in the same manner, except that the common carotid arteries were not ligated. After surgery, the wounds were sutured, and the rats were placed on a homeothermic blanket until they recovered from the anesthesia.

### Cerebral Blood Flow

The measurement of blood flow in the hippocampus was performed as described previously (Jian et al., 2013). After anesthetization, rats were fixed in a stereotactic frame with a midsagittal incision on top. In order to detect blood flow in the hippocampal CA1 region (anteroposterior = 4.8 mm, mediolateral = ± 2.5 mm, and dorsoventral = −3.5 mm), a skull hole was made above this area on the left side, and a 0.45-mm-diameter laser Doppler probe was used to drill into the hippocampus from the hole. When stable cerebral blood flow was observed, hippocampal blood flow was continuously recorded for 5 min using Perisoft software. A similar measurement procedure was performed immediately after completion of 2VO or sham surgery. After the measurement was completed, the probe was pulled out, and the wound was sutured. The preoperative measurement value was used as the baseline, and the results were expressed as a percentage of the second measurement value to the baseline value.

### Stereotaxic Injection

After anesthetization, rats were placed in a stereotaxic head holder. Solutions of the virus were injected bilaterally into the hippocampal CA1 region (anteroposterior = 4.8 mm, mediolateral = ± 2.5 mm, and dorsoventral = −3.5 mm) with an injection rate of 0.5 μl/min (Shen et al., 2019). The effect of viral transfection was evaluated using western blotting at different time points after transfection.

### MWM Test

The MWM is a classical test of spatial learning and memory for rodents (Redish and Touretzky, 1998; Yu et al., 2019). The MWM consisted of a circular pool (150 cm in diameter and 60 cm in height) filled with opaque water to a depth of 32 cm at a temperature of 20 ± 1°C. The maze was equally divided into four quadrants by four signs on the pool. A platform (9 cm in diameter and 30 cm in height) was placed in one quadrant and was invisible in the water. The pool was located in a dimly lit room surrounded by several orientation cues. Each rat was given four trials per day for five consecutive days. Rats were randomly placed into the pool from a different quadrant in each trial, facing the wall of the maze. The time for the rats to find the hidden platform was recorded if it was less than 60 s. However, if the time exceeded 60 s, the latency time was recorded as 60 s. All rats were placed on the platform to observe their surroundings for 20 s after each trial. On the sixth day, each rat was subjected to a probe trial for 60 s in the maze in which the platform was removed. The time the rats swam in the target quadrant (where the platform had been placed) was recorded.

### NOR Test

The NOR test was performed as described previously (Bevins and Besheer, 2006). The test consisted of a test box (white square box, 65 cm × 45 cm × 40 cm) and two sets (two per set) of different objects. The test object set A contained two identical white printed porcelain cups with a base diameter of 6.5 cm and a height of 10 cm; while the B set was made of two identical cylindrical transparent glass bottles with a bottom diameter of 5 cm and a height of 8 cm. The test environment was quiet and dark, and the light in the test box was even without shadow. In the first stage (adaptation), no objects were placed in the box. A rat was placed in the test box with its back to the box and allowed to move by itself for 10 min. The next day, two identical objects (AA) were placed symmetrically in the box (9 cm from the long axis and 10 cm from the short axis). The rat was placed with its back to the objects from the same distance point between the two objects and allowed to move by itself for 10 min. Then the rat was returned to its home cage. After 1 h, two different objects (AB) were placed in the box in the same position as described above, and the rat was left to explore the box for 5 min. The stopwatch software (Time Left 3) was used to record the exploration time of the old object (A) and the novel object (B) when different objects (AB) were placed. A discrimination ratio (DI) of exploring the novel object was calculated, expressed as DI = N/(N + F), where N was the time for exploring the novel object and F was the time for exploring the old object.

### Measurement of BBB Permeability

The permeability of BBB was evaluated using the EB extravasation technique (Huang et al., 2018; Ni et al., 2018; Luh et al., 2019). Rats were injected with 2% EB (Sigma, 4 ml/kg) through the tail vein. After 2 h, the rats were deeply anesthetized and infused with 50 ml heparinized saline through the left ventricle for 15 min. The hippocampal specimens were removed and immersed in formamide (3 ml/100 mg) at 60°C for 24 h and then centrifuged at 15,000 *g* for 30 min at 4°C. Spectrophotometric determination of extravasated EB in the supernatant was assayed at 620 nm.

### TEM

After anesthetization, rats were perfused with saline for 1 min and subsequently with 5% glutaraldehyde and 4% paraformaldehyde for 4 min. Brain tissues of the hippocampal CA1 region (1 mm × 1 mm × 1 mm) were removed and postfixed at 4°C. Then the tissues were dehydrated in gradient ethanol and embedded in epoxy resin. Sections (80 nm) were cut from the embedded specimens with an ultrathin slicer (Leica EM UC7, Germany), placed on copper grids, stained with lead citrate and uranyl acetate, and observed using a Tecnai-G220-TWIN TEM (FEI, United States). Quantitative analysis of vesicles of six comparable-sized vessels in each rat was performed.

### Immunofluorescence Staining

After perfusion, the brains were removed and fixed with 4% PFA at room temperature for 1 h, followed by immersion in 30% sucrose. The brains were then cryopreserved in OCT and sectioned in a cryostat. After blocking with goat serum, the sections were incubated with anti-Mfsd2a primary antibody (species: rabbit; 1:50; Abcam) overnight in a dark chamber. The next day, fluorescent secondary antibody (FITC-labeled goat anti-rabbit antibody) was added, followed by blocking with goat serum. The blocking solution was decanted, and the sections were incubated with anti-CD31 primary antibody (species: mouse; 1:100; Abcam) overnight in the dark. The following day, fluorescent secondary antibody (Cy3-labeled goat anti-mouse antibody) was added. The brain sections were visualized using a fluorescence microscope (OLYMPUS BX53, Japan).

### Western Blotting

After anesthetization, the hippocampi of rats were rapidly removed. Total proteins were extracted using RIPA lysis buffer, and the protein concentration was estimated using the BCA protein assay. Protein samples (40 μg) were fractionated with 10% sodium dodecyl sulfate-polyacrylamide gels, transferred to nitrocellulose membranes, and blocked for 2 h in 5% skimmed milk at room temperature. Next, the membranes were incubated with primary antibodies against Mfsd2a (1:1,000, Abcam), ZO-1 (1:500, Santa Cruz, CA, United States), occludin (1:500, Abcam), claudin-5 (1:500, Bioss), or GAPDH (1:10,000; Abcam) at 4°C overnight. The blots were washed three times with TBST and incubated for 30 min in horseradish peroxidase-conjugated goat anti-mouse secondary antibody (1:10,000; ASPEN) or goat anti-rabbit secondary antibody (1:10,000; ASPEN). Specific protein bands were visualized using the chemiluminescence detection kit (Amersham). The band intensities were measured using Band Scan 5.0 software (Alpha Innotech Corp.).

### Statistical Analysis

The statistical analyses were done by using SPSS for Windows (version 24). Data were presented as mean ± SEM. Differences in escape latency were analyzed with a two-way repeated-measures ANOVA followed by the *post hoc* Bonferroni test for multiple comparisons. The significance of differences between two and three or more groups was determined using one-way ANOVA followed by the Bonferroni *post hoc* test, the Student’s *t*-test, or non-parametric tests. Statistical significance was defined as *P* < 0.05.

## Results

### Expression of Mfsd2a Protein Was Downregulated in the Hippocampal CA1 Region of CCH Rats

**FIGURE 1:**
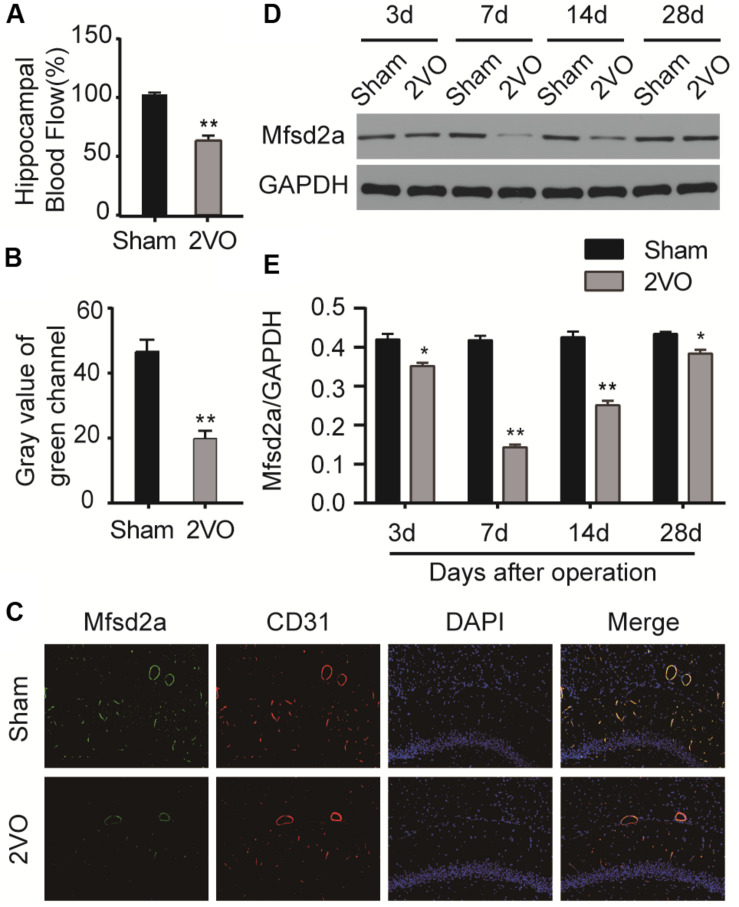
The blood flow and the time course of changes in major facilitator superfamily domain-containing protein 2a (Mfsd2a) in the hippocampus following chronic cerebral hypoperfusion (CCH). **(A)** Changes in hippocampal blood flow (percentage of the postoperative hippocampal blood flow value to the baseline hippocampal blood flow value). **(B)** Gray value of the green channel. **(C)** Immunofluorescence staining for Mfsd2a (green) and CD31 (red) in the microvascular endothelial cells of the hippocampus from sham and CCH rats at 7 days after 2VO operation. The nuclei were labeled with DAPI (blue). (**D**) Western blot band. (**E**) Quantification of the relative expression of Mfsd2a. *P < 0.05, **P < 0.01, vs. the sham group; *n* = 6 per group. Scale bar = 100 μm.

Preoperative measurements of hippocampal blood flow in each group were used as their baseline values. The hippocampal blood flow decreased significantly after 2VO surgery but did not change obviously after sham surgery. There were significant differences in hippocampal blood flow changes between the two groups (*P* < 0.01, Figure 1A).

Western blot was performed to measure the expression levels of Mfsd2a in the hippocampal CA1 region of rats at different time points after 2VO surgery. The expression of Mfsd2a protein decreased from postoperative day 3 (*P* < 0.05) and reached the lowest level on day 7 (*P* < 0.01, Figures 1D,E). The expression level began to recover on day 14 (*P* < 0.01) but remained lower than that of the sham group on day 28 (*P* < 0.05). In addition, the results of immunofluorescence staining also confirmed that the fluorescence intensity of Mfsd2a in the 2VO group was lower than that of the sham group (Figures 1B,C, *P* < 0.01). These results suggest that the expression of Mfsd2a protein in the hippocampal CA1 region was downregulated after CCH.

### Overexpression of Mfsd2a Reversed Learning and Memory Deficits in CCH Rats

**FIGURE 2:**
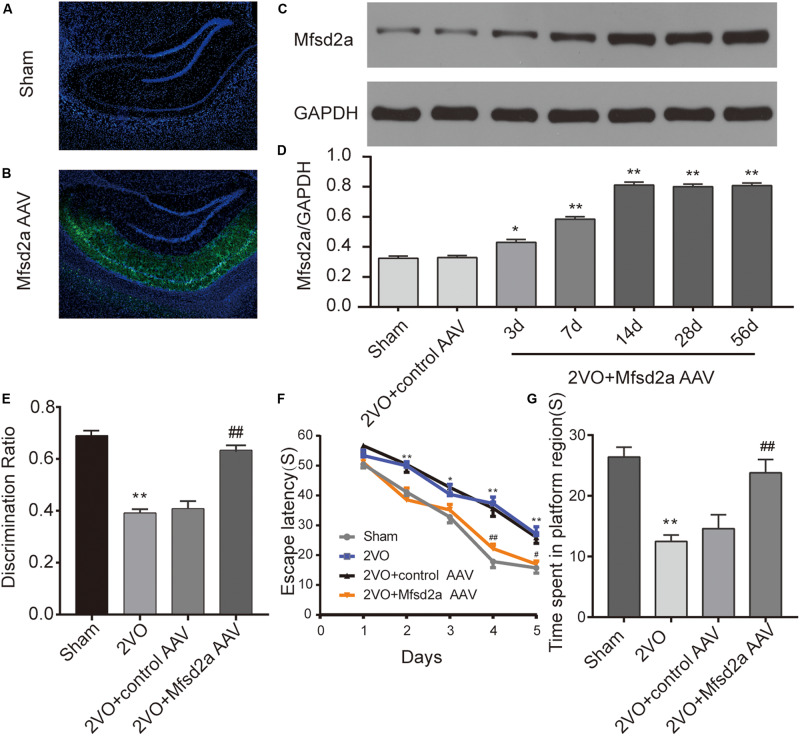
Effect of Mfsd2a overexpression on learning and memory of rats. **(A,B)** Representative immunofluorescence images of the rat hippocampus at 14 days after viral transfection: **(A)** sham group; **(B)** 2VO + Mfsd2a AAV group: stereotaxic injection of the Mfsd2a adeno-associated virus (AAV-CMV-Mfsd2a-ZsGreen) targeting the rat CA1 region. The nuclei were labeled with DAPI (blue). **(C)** Western blot band of Mfsd2a in the hippocampus of rats at different time points after transfection with the AAVs. **(D)** Quantification of the relative expression of Mfsd2a. *P < 0.05, **P < 0.01, vs. the sham group; *n* = 6 per group. **(E)** Discrimination ratio scores of each group in the novel object recognition test. **(F)** The escape latency on days 1–5 in the Morris water maze. **(G)** Time spent in the target quadrant during the spatial probe test. *P < 0.05, **P < 0.01, vs. the sham group; ^#^*P* < 0.05, ^##^*P* < 0.01, vs. the 2VO + control AAV group; *n* = 9 per group. Scale bar = 500 μm.

The virus was successfully transfected into the hippocampus of rats via stereotaxic injection (Figures 2A,B). To validate the effect of viral transfection, western blotting technology was used to evaluate the expression of Mfsd2a protein in the rat hippocampus at different time points after transfection with the Mfsd2a AAVs. The data showed that the expression of Mfsd2a in the 2VO + Mfsd2a AAV group remained at a high level from day 14 to day 56 post transfection (*P* < 0.01, Figures 2C,D).

The results of the MWM test showed that the escape latency of rats was significantly shortened in all groups as training progressed [*F*(4,128) = 159.83, *P* < 0.01], but there were significant differences across the four groups [*F*(3,32) = 21.13, *P* < 0.01, Figure 2F). Bonferroni *post hoc* test showed that from day 2 of training, rats in the 2VO group required a longer time to locate the platform than did the sham rats (at days 2, 4, and 5, *P* < 0.01; at days 3, *P* < 0.05). Between days 3 and 5 of training, the rats in the 2VO + Mfsd2a AAV group required substantially less time to locate the platform compared with the 2VO + control AAV rats, at the corresponding time points (at days 3 and 5, *P* < 0.05; at day 4, *P* < 0.01).

In the probe trial where the platform was removed, memory was evaluated by measuring the time spent in the target quadrant (Figure 2G). We observed that the 2VO rats spent significantly less time in the target quadrant than the sham rats (*P* < 0.01). The time spent in the target quadrant by the 2VO + Mfsd2a AAV rats was significantly increased, in comparison to that spent by the 2VO + control AAV rats (*P* < 0.01).

The results of the NOR test showed that rats in the 2VO group had lower DI scores than the sham rats (*P* < 0.01, Figure 2E), indicating a recognition memory impairment following 2VO surgery. However, the transfection with the Mfsd2a AAVs significantly improved the DI scores (2VO + Mfsd2a AAV group vs. 2VO + control AAV group, *P* < 0.01).

### Overexpression of Mfsd2a Attenuated BBB Leakage in CCH Rats

**FIGURE 3:**
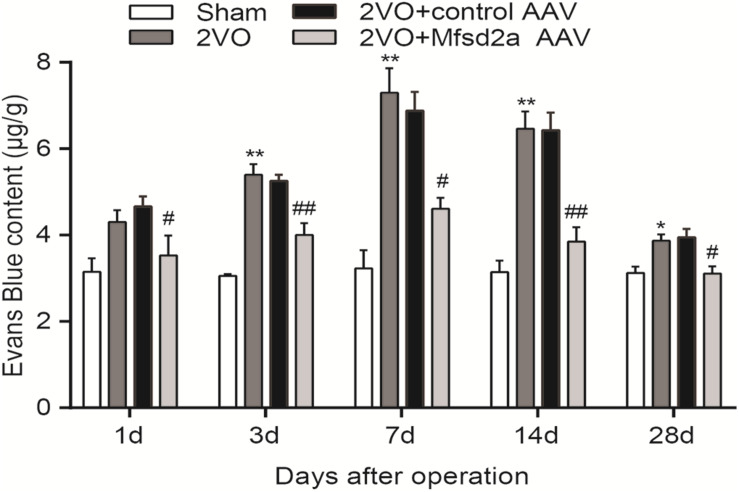
Time course of Evans blue content in the hippocampus of rats in each group after 2VO surgery. *P < 0.05, **P < 0.01, vs. the sham group at the corresponding time point; ^#^*P* < 0.05, ^##^*P* < 0.01, vs. the 2VO + control AAV group; *n* = 4 per group.

To determine the effect of Mfsd2a overexpression on CCH-induced BBB damage, we quantified the amount of EB in the hippocampal CA1 region using colorimetric analysis. The results showed that compared with the sham rats, EB leakage in the 2VO group started increasing from day 3 after the 2VO procedure (*P* < 0.01) and reached its peak on day 7 (*P* < 0.01). The increase remained significant on day 14 (*P* < 0.01), and by day 28, EB leakage remained higher than that in the sham group (*P* < 0.05, Figure 3). In contrast, the 2VO + Mfsd2a AAV rats exhibited significantly reduced EB leakage at all time points (vs. the 2VO + control AAV group, at days 1, 7, and 28, *P* < 0.05; at days 3 and 14, *P* < 0.01).

### Effect of Mfsd2a Overexpression on BBB Tight Junctions and Vesicular Transcytosis in the Rat Hippocampal CA1 Region

**FIGURE 4:**
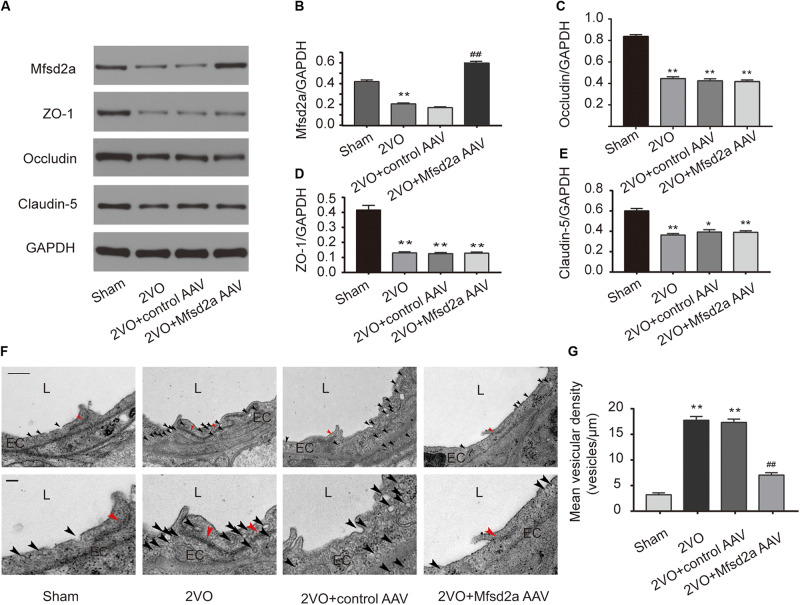
Effect of Mfsd2a overexpression on BBB tight junctions and vesicular transcytosis in the rat hippocampal CA1 region. **(A–E)** The expression of BBB permeability-related proteins, including Mfsd2a, ZO-1, claudin-5, and occludin in each group. *P < 0.05, **P < 0.01, vs. the sham group; ^#^*P* < 0.05, ^##^*P* < 0.01, vs. the 2VO + control AAV group; *n* = 6 per group. **(F)** Representative microphotograph of the ultrastructure of BBB in the hippocampal CA1 region of rats. Scale bar = 500 nm (the upper four pictures); scale bar = 150 nm (the lower four pictures). **(G)** Quantification of vesicular density of six comparable-sized vessels (4–5 μm lumen) in each rat. *P < 0.05, **P < 0.01, vs. the sham group; ^#^*P* < 0.05, ^##^*P* < 0.01, vs. the 2VO + control AAV group; *n* = 3 per group. The black arrow refers to vesicles in endothelial cells, and the red arrow refers to tight junctions. L, lumen; EC, endothelial cell.

We used western blotting to examine the expression of Mfsd2a and tight junction-associated proteins in the hippocampal CA1 region 7 days after the 2VO procedure. The expression levels of Mfsd2a, ZO-1, occludin, and claudin-5 in the hippocampal CA1 region of rats in the 2VO group were downregulated compared to those in the sham group (*P* < 0.01, Figures 4A–E). Compared with the 2VO + control AAV group, the expression of Mfsd2a significantly increased in the 2VO + Mfsd2a AAV group (*P* < 0.01), whereas no significant differences were observed in the expression levels of ZO-1, claudin-5, and occludin proteins (*P* > 0.05).

In addition, we utilized TEM to observe the changes in the BBB ultrastructures in the hippocampal CA1 region following Mfsd2a overexpression in CCH rats (Figures 4F,G). The results showed that the vesicular densities in the brain microvascular ECs were significantly higher in rats of the 2VO and 2VO + control AAV groups than in the sham group (*P* < 0.01). However, the vesicular density was significantly lower in rats of the 2VO + Mfsd2a AAV group than in the 2VO + control AAV group (*P* < 0.01). The tight junction structures were not significantly different among the four groups.

## Discussion

We have identified that the expression level of Mfsd2a protein is reduced in the hippocampus of CCH rats, leading to enhanced vesicle transcytosis and resulting in high permeability of BBB. The recombinant AAV (overexpressing Mfsd2a) upregulated the expression of Mfsd2a protein in the hippocampus of CCH rats, inhibited the active vesicle transcytosis, and ameliorated cognitive impairment of CCH rats. These findings reemphasize the importance of the BBB in cognitive impairment and for the first time elucidate the role of Mfsd2a in the regulation of BBB permeability in CCH rats, indicating that not only is paracellular transport involved in this process but also that vesicle transcytosis cannot be neglected.

The BBB is critical for maintaining the normal function of the central nervous system. It limits the entry of blood-borne neurotoxins into the brain and helps eliminate harmful substances produced internally, thereby avoiding neuronal injury and sustaining a stable brain microenvironment (Zlokovic, 2011; Iadecola, 2013). BBB damage is a key pathophysiological factor in CCH-induced cognitive impairment (Ueno et al., 2002; Chen et al., 2015; Yin et al., 2015). Therefore, protection of the BBB is believed to be a promising strategy to improve cognitive function after CCH (Edrissi et al., 2016; Lee et al., 2017). The expression of tight junction-associated proteins such as ZO-1, claudin-5, and occludin decreased after CCH, and regulation of these proteins can rectify the CCH-induced BBB hyperpermeability, thereby improving cognitive function (Hawkins et al., 2004; Edrissi et al., 2016; Lee et al., 2017). However, these studies explored the role of tight junctions as one of the two key factors in maintaining BBB permeability but did not describe the role of another key factor, vesicle endocytosis (Haseloff et al., 2015). The extremely low rate of vesicular transcytosis is vital for maintaining the barrier function of the BBB (Haseloff et al., 2015). The expression of Mfsd2a (Ben-Zvi et al., 2014), the key protein to maintain this effect, is decreased under some pathological conditions, leading to an increase in the number of vesicles and an active transcytosis, which in turn leads to a significant increase in BBB permeability and aggravation of nerve function damage (Andreone et al., 2017; Yang Y. R. et al., 2017). Consistent with this, our results also showed that CCH caused a decrease in Mfsd2a expression and an enhancement of vesicle transcytosis. This may be related to pericytes. The expression of Mfsd2a is regulated by pericytes, and knockout of pericytes can cause the disappearance of Mfsd2a (Ben-Zvi et al., 2014). Recent evidence has shown that pericytes in the BBB are significantly reduced in the coverage of endothelial cells after CCH (Liu et al., 2019). However, the researchers did not detect Mfsd2a expression at the time.

Major facilitator superfamily domain-containing protein 2a is a novel mammalian major facilitator superfamily domain protein, first identified in 2008 (Angers et al., 2008). It was found by chance in a study that Mfsd2a was highly expressed in brain tissues (Ben-Zvi et al., 2014). Mfsd2a has recently been identified as an important component of BBB formation and integrity. Ablation of Mfsd2a results in increased BBB leakage from embryo to adult without disruption of tight junctions (Ben-Zvi et al., 2014). Since then, Mfsd2a has been involved in the study of neurological diseases related to BBB integrity. For example, in the early stage of cerebral hemorrhage (Yang Y. R. et al., 2017; Zhao et al., 2020) and cerebral infarction (Andreone et al., 2017), the expression of Mfsd2a decreases, and upregulating its expression can reduce neurological damage. In the cognitive impairment caused by CCH, BBB dysfunction can trigger neuroinflammation and oxidative stress, cause brain cell edema and neuron apoptosis, increase amyloid beta production, and decrease its clearance, and the toxic effect of amyloid beta further aggravates BBB dysfunction, finally leading to cognitive impairment (Cai et al., 2018; Cockerill et al., 2018). Our results showed that upregulation of Mfsd2a could reduce the BBB damage caused by CCH, partially break this vicious circle, and improve the cognitive dysfunction of CCH rats.

There may be some possible limitations in this study. To validate the effect of viral transfection, western blotting technology was used to evaluate the expression of Mfsd2a protein in the rat hippocampus after transfection with the Mfsd2a AAVs. If double staining of sections for Mfsd2a and CD31 and colocalization analysis were conducted in the research, then the result could be more convincing. In the present experiment, the effect of Mfsd2a gene deletion (such as a gene knockout model) on CCH-induced cognitive impairment was not evaluated, and the relevant mechanisms were not further explored. In future studies, we will try to improve experimental animals and methods to further explore the role of Mfsd2a in cognition. In addition, the role of Mfsd2a in other neurological diseases closely related to BBB, such as Parkinson’s disease, epilepsy, and intracranial infection, is also worthy of investigation.

## Conclusion

In conclusion, this is the first report exploring the relationship between cognition and the vesicle endocytosis. Mfsd2a alleviates CCH-induced BBB damage by inhibiting vesicular transcytosis, thereby improving spatial learning and memory impairment in CCH rats. Our results provide new evidence on the amelioration of cognitive function via BBB protection and present novel targets for the prevention and treatment of CCH-related diseases, such as VCI and Alzheimer’s disease.

## Data Availability Statement

The datasets generated for this study are available on request to the corresponding author.

## Ethics Statement

The animal study was reviewed and approved by the Animal Ethics Committee of the Medical School of Wuhan University.

## Author Contributions

ChQ and HS were involved in the study design, performed the study, and drafted and revised the manuscript. JS was involved in data analysis. LX was involved in data analysis and performed the animal study. YL, CjQ, and TL performed the animal study. JZ was involved in the study design and obtaining funding. All authors contributed to manuscript revision and, read and approved the submitted version.

## Conflict of Interest

The authors declare that the research was conducted in the absence of any commercial or financial relationships that could be construed as a potential conflict of interest.

## Abbreviations

AAV: adeno-associated virus
BBB: blood–brain barrier
CCH: chronic cerebral hypoperfusion
EB: Evans blue
EC: endothelial cell
Mfsd2a: major facilitator superfamily domain-containing protein 2a
MWM: Morris water maze
NOR: novel object recognition
VCI: vascular cognitive impairment
TEM: transmission electron microscopy
2VO: bilateral common carotid artery occlusion
ZO-1: zonula occludens-1.
